# The more positive intergroup contacts you have, the less LGBTQ+ conspiracies beliefs you will report: The role of knowledge, anxiety, and empathy

**DOI:** 10.1111/bjso.12866

**Published:** 2025-02-15

**Authors:** Sara Panerati, Marco Salvati

**Affiliations:** ^1^ Department of Human Sciences University of Verona Verona Italy

**Keywords:** intergroup anxiety, intergroup contact, intergroup empathy, intergroup knowledge, LGBTQ+ conspiracy beliefs

## Abstract

Conspiracy theories and beliefs against LGBTQ+ people are a recurrent theme in the political agenda, depicting them as evil actors in a larger plot, seeking to undermine societal norms, institutions, and traditional values. Lessening LGBTQ+ conspiracy beliefs is crucial to reaching more social equality, and intergroup contact might represent a useful strategy. Study 1 (*N* = 253) investigated the associations of the quantity of direct contact with LGBTQ+ people, the quality of such contacts, and their interactive role with LGBTQ+ conspiracy beliefs. Taking a step forward, Studies 2 (*N* = 512) and 3 (*N* = 529) investigated, correlationally and experimentally, respectively, the relationship between the quality of contact with LGBTQ+ individuals and LGBTQ+ conspiracy beliefs, exploring the mediating associations of intergroup knowledge, empathy, and anxiety. Results consistently suggested that a higher quantity of direct contacts with LGBTQ+ people is negatively associated with LGBTQ+ conspiracy beliefs. Furthermore, positive contact was associated with lower conspiracy beliefs against LGBTQ+ people, with these associations being either partially (Study 2) or fully (Study 3) mediated by intergroup empathy. Overall, these findings highlight the importance of fostering positive intergroup interactions and enhancing empathy as strategies to combat harmful conspiracy beliefs about marginalized groups.

## INTRODUCTION

The Council of the European Commission against Racism and Intolerance (ECRI) annual report (2024) pointed out unceasing discrimination and hostility against Lesbian, Gay, Bisexual, Transgender, Queer, and Intersexual (LGBTQ+) people, even though EU member states agreed that ‘Gender equality is a core value of the EU, a fundamental right and key principle of the European Pillar of Social Rights’ (European Commission: Directorate‐General for Justice and Consumers, 2024). Despite some positive achievements (e.g., increasing protections for same‐gender couples and banning so‐called ‘conversion therapies’), relentless opposition has been a recurring theme in all legal debates concerning issues of sexual orientations, gender identities, and gender expressions in the UN member states. This resistance and derogation are so pervasive that it is difficult to pinpoint consistent global positive trends, as progress in these categories is also uneven across and within regions (ILGA World, 2022).

Among the factors that still contribute to maintaining LGBTQ+ as disadvantaged groups, the ECRI annual report (2024) pointed out the undermining role of hate speech by politicians and hate‐motivated violence directed against the LGBTQ+ community. Particularly, conspiracy theories and beliefs about LGBTQ+ people are a recurrent theme in the political agenda as justifications to fight against the passing of protective laws (e.g., regarding the promotion of anti‐discrimination policies) and to affect public opinion negatively (Salvati, Pellegrini, De Cristofaro, Costacurta, & Giacomantonio, 2024; Wardawy‐Dudziak, 2024). These inflame and preserve stereotypes, stigma, and prejudice revolving around them contributing to hindering the rights and well‐being of LGBTQ+ people (Jolley et al., 2020; Klein & Nera, 2020). Despite the prevalence and known negative impact of conspiracy beliefs both on attitudes (e.g., Jolley & Douglas, 2014) and behaviours (e.g., Lewandowsky et al., 2013), there is a lack of research examining possible strategies to reduce this phenomenon (e.g., Cheso et al., 2024). According to research, conspiracy beliefs might be extremely difficult to change directly since providing counter‐factual evidence against them can be interpreted as a product of the conspiracy theories themselves (Sunstein & Vermeule, 2009, see also Jolley & Douglas, 2014, Jolley & Douglas, 2017). Therefore, finding strategies and interventions to lessen (LGBTQ+) conspiracy beliefs is crucial to reach more social equality (Brown, 2005). In this regard, intergroup contact may represent one of the effective strategies for reducing conspiracy theories (Jolley et al., 2023) and increasing the acceptance of gay, lesbian, bisexual people (Basow & Johnson, 2000; Hinrichs & Rosenberg, 2002) and trans people (Hill & Willoughby, 2005).

### Conspiracy theories and beliefs against LGBTQ+

Although the conspiracy beliefs have raised global attention due to the severe and undesirable social consequences they produce (e.g., antivaccine movements; Ullah et al., 2021), research on LGBTQ+ conspiracy beliefs is still quite rare and literature on the topic is still widely unexplored (Salvati, Pellegrini, De Cristofaro, Costacurta, & Giacomantonio, 2024). Despite general conspiracy beliefs and LGBTQ+ conspiracy beliefs sharing some similarities (such as the presence of an elite ‘against’ the majority of heterosexual people, the secrecy dimension and the aspect of power; Douglas et al., 2019) LGBTQ+ conspiracy beliefs seem to refer to a specific phenomenon with unique characteristics (Salvati, Pellegrini, De Cristofaro, Costacurta, & Giacomantonio, 2024), for instance concerning content‐specific beliefs and targeting a particular subgroup of people (Gkinopoulos et al., 2024).

At their roots, LGBTQ+ conspiracy beliefs perpetuate the idea that LGBTQ+ people represent a lobby that constitutes a threat to social order and morality in our society (Salvati, Pellegrini, De Cristofaro, & Giacomantonio, 2024; Trappolin, 2022). They are seen as actors in a vast conspiracy that aims to threaten institutions, societal norms and traditional values (Salvati, Pellegrini, De Cristofaro, & Giacomantonio, 2024). The LGBTQ+ conspiracy beliefs often rely on the idea that LGBTQ+ deviate from heteronormativity, hence claiming the existence of a hierarchical societal system that encompasses heterosexuality and binary gender identity as normal and natural, and in turn, defining boundaries of what is acceptable concerning heterosexuality and gender identity (Scandurra et al., 2021). Since LGBTQ+ people do not conform to these norms, they are depicted as a threat to the status quo and values of the (traditional) society, for instance, by triggering conflicts between the sexes, fostering hostility to traditional parenthood and destroying the ‘sacred institution’ of the family (Mouafo et al., 2024). Hence, in a context where heteronormativity is strongly enforced, the traditional social structure and values of the society could be perceived as threatened by the LGBTQ+ people (Mackey, 2024; Tjipto et al., 2019). This, in turn, can lead to embracing LGBTQ+ conspiracy beliefs which have been associated with detrimental consequences, such as supporting social exclusion (Graeupner & Coman, 2017), intergroup violence and discrimination against this community (Mouafo et al., 2024).

Such a conspiracy narrative paves the way to intergroup hostility and conflict; in fact, it leads (majority) people to feel vulnerable and makes it easy to take advantage of this reaction and push for radical political actions and division in our society (Moskalenko & Romanova, 2022). This, in turn, seems to lead to an increase in the distance towards the LGBTQ+ group (Gkinopoulos et al., 2024), a decrease in support for LGBTQ+ civil rights and reduce the intentions to support these communities through collective actions (Salvati, Pellegrini, De Cristofaro, Costacurta, & Giacomantonio, 2024).

### Intergroup contact as a strategy to reduce conspiracy beliefs

Thus far, research investigating the psychology of conspiracy beliefs mainly focuses on a twofold aim (Jolley et al., 2023). On the one hand, the focus revolved around pinpointing the individual factors more closely associated with the probability of adopting conspiracy narratives (e.g., distrust in authority, low levels of interpersonal trust, and cynicism; see Douglas et al., 2019 for a review). On the other hand, studies have also looked at the consequences of holding these beliefs revealing their detrimental influence on running society (see Jolley et al., 2022, for a review).

However, little is known about strategies to reduce conspiracy beliefs (Jolley et al., 2023). According to research, conspiracy beliefs might be extremely difficult to change straightforwardly: believers in conspiracy theories may overlook factual information, selectively pick certain pieces of information or assume that fact‐checkers are part of the conspiracy theories themselves (Tingley & Wagner, 2017). In this regard, Freelon (2024) highlighted that conspiracy beliefs and prejudice could have a closer conceptual and normative relationship than previous studies have shown: they could manifest in the same people, produce similar detrimental effects against the outgroup and benefit from the same interventions. Hence, research started to underline the importance of finding alternative approaches to reduce conspiracy beliefs and one of the possible strategies can be identified in intergroup contact (Allport, 1954; Jolley et al., 2023). In a meta‐analytical work, Pettigrew and Tropp (2006) across hundreds of studies pointed out that intergroup contact was usually associated with a decrease in intergroup prejudice (*r* = .21). The underlying premise of this theory was that interactions between ingroup and outgroup members could lessen the prejudices that ingroup members held against the outgroup under certain conditions (i.e., equal status, common goals, intergroup cooperation, and institutional support; Kanamori et al., 2022). Moreover, intergroup contact has been shown to prompt several outcomes, such as positively affecting the general evaluations or emotions towards the outgroup (Capozza et al., 2013), leading to changes in political attitudes and policy support (Cakal et al., 2011), and less extreme political attitudes (Stringer et al., 2009). But how can intergroup contact decrease conspiracy beliefs?

Many studies have found that intergroup contact is associated with reduced prejudice (Wagner et al., 2020). Nevertheless, some studies found the opposite association (e.g., Green et al., 2010; Putnam, 2007), suggesting that further processes may determine whether contact may benefit or harm the relations between groups. Possible explanations concerning the differential effects of contact opportunities on prejudice are the valence (i.e., quality) and frequencies (i.e., quantity) of intergroup contact people may engage in (Kotzur & Wagner, 2021). In this regard, Jolley et al. (2023) provided a first attempt to investigate correlationally and experimentally (via imaginative contact) the relationship between positive contact and the reduction in conspiracy theorizing against immigrants and Jewish people. Their findings pointed out that positive contact with immigrants and Jewish people was associated with lower belief in outgroup‐directed conspiracy theories.

Furthermore, early intergroup contact theorists generally believed that intergroup contact may improve ingroup knowledge about the outgroup by providing the opportunity to learn from each other (Allport, 1954). This, in turn, may reduce prejudice (Cheso et al., 2024). Despite the great attention given to the associations between intergroup knowledge and prejudice reduction (see Pettigrew & Tropp, 2008), little attention has been given in the literature to the association between intergroup knowledge and conspiracy beliefs (Uscinski et al., 2016). Sallam et al. (2020) pointed out that COVID‐19‐related conspiracy beliefs were associated with a lower level of knowledge and that believing that the disease was part of a conspiracy theory was strictly related to misinformation about the availability of a vaccine and the use of antibiotics for COVID‐19 treatments.

Taking a step forward, another possible mediator between intergroup contact and prejudice revolves around perspective‐taking and empathy (Bobba & Crocetti, 2022; Pettigrew & Tropp, 2008). In this regard, Hodson et al. (2018) argued that contact may help people change the way they think about the world: instead of relying on broad and group‐based categories, they may switch to a more individualized and systematic information processing when encountering outgroup members who do not simply fit into an existing category (Fiske & Neuberg, 1990). Therefore, intergroup contact can be seen as a way to recategorize ingroup and outgroup members, which may enable one to understand more outgroup members' issues and adopt their perspective (Vescio et al., 2003). In this regard, a recent work (Jolley et al., 2025), investigated whether parasocial intergroup contact (i.e., observing an outgroup member on the media) between cisgender and transgender people was associated with lower transgender conspiracy theories via (an enhanced) perspective‐taking concerning transgender people. Their findings demonstrated that although positive intergroup contact did not directly reduce conspiracy beliefs, positive parasocial contact indeed increased perspective‐taking which, in turn, was associated with lower transgender conspiracy beliefs. The perspective‐taking process, in turn, is closely related to intergroup empathy (i.e., the emotional response to the emotion(s) experienced by members of the outgroup; Capozza et al., 2013) which has been seen as playing a fundamental role in improving intergroup relations (Stephan & Finlay, 1999). For instance, it has been seen to strengthen prosocial behaviours (Johnston & Glasford, 2018), enhance intergroup attitudes and reduce intergroup bias (Dovidio et al., 2010), improve sensitivity to hate speech and support for hate speech prohibition, and reduce intentions to use derogatory language in the future (Soral et al., 2022).

Finally, another major factor playing a role in the relationship between intergroup contact and prejudice reduction is strictly related to the perception of the outgroup as a threat. In this regard, researchers have been testing the contact–prejudice association by focusing on the perception of the outgroup as a threat eliciting intergroup anxiety (i.e. the feelings of uneasiness and awkwardness in anticipation of interacting with outgroup members; Capozza et al., 2013). Particularly, research repeatedly demonstrated that intergroup contact typically reduces intergroup anxiety (Paolini et al., 2004; Stephan et al., 2002; Voci & Hewstone, 2003). In other words, favourable contact experiences are usually associated with lower intergroup anxiety, which in turn is related to lower levels of prejudice. This is true when the contact happens in a face‐to‐face context but also indirectly through imagined contact or via extended contact (Kanamori et al., 2022; Stephan, 2014). Although there is strong evidence linking outgroup threat to prejudice, still more research on the specific mechanisms involved (e.g., the role of conspiracy thinking and anxiety) is needed (Murphy et al., 2024).

To summarize, intergroup contact seems to decrease outgroup prejudice through three main (mediational) paths, namely, increasing intergroup knowledge, increasing intergroup empathy and perspective‐taking, and reducing intergroup anxiety (Pettigrew & Tropp, 2008). In addition to these more traditional associations, intergroup contact could represent a useful approach not only for addressing prejudice but also for changing more distal outcomes (Hodson et al., 2018), such as conspiracy beliefs (Jolley et al., 2023).

#### The current research and hypotheses

Despite the literature on intergroup contact showing a plethora of evidence regarding the positive effect of intergroup contact in reducing intergroup prejudice (Pettigrew & Tropp, 2006, 2008), to the best of our knowledge, works grounded on intergroup contact that addressed factors decreasing LGBTQ+ conspiracy beliefs are novel. Hence, expanding on this previous evidence on the promising role of intergroup contact on conspiracy theories, in Study 1, we investigate a moderation model including the quantity of direct contacts with LGBTQ+ people, the quality of such contacts and their interaction, as predictors of LGBTQ+ conspiracy beliefs by also controlling for a modern measure of sexual prejudice. We hypothesized that higher contact with LGBTQ+ people would be associated with lower adherence to LGBTQ+ conspiracy beliefs (Hypothesis 1) and that the more people would perceive such contacts as positive, the less they would adhere to LGBTQ+ conspiracy beliefs (Hypothesis 2). Furthermore, we expected that the relationship between the quantity of LGBTQ+ contacts and LGBTQ+ conspiracy beliefs would be qualified by the levels of quality of LGBTQ+ contacts (Hypothesis 3). Specifically, for people with a higher number of direct contacts with LGBTQ+ people, the quality of such contacts might be less relevant in predicting LGBTQ+ conspiracy beliefs, since in probabilistic terms it is statistically more likely that such contacts will be prominently positive. Instead, for people having a lower number of direct contacts with LGBTQ+ people, the quality of such contacts might have a greater impact on LGBTQ+ conspiracy beliefs, in the direction that people with worse contact quality with LGBTQ+ people would report more LGBTQ+ conspiracy beliefs, compared with people with better contact quality with LGBTQ+ people.

Study 2 took a step forward by investigating the potential processes that might contribute to explaining the relationship between the higher quality of LGBTQ+ contacts and the lower adherence to LGBTQ+ conspiracy beliefs. Based on the theoretical framework of contact theory (Allport, 1954), we selected the three main mediators explored in previous literature on the relationship between intergroup contact and prejudice, that are the increased knowledge about the outgroup; the reduced intergroup anxiety, and the increased intergroup empathy (Pettigrew & Tropp, 2006, 2008).

Specifically, the hypotheses of this second correlational study were that the more people would report positive LGBTQ+ contacts, the less they would show LGBTQ+ conspiracy beliefs, through an indirect association of (a) an increased level of knowledge about LGBTQ+ group (hypothesis 1); (b) a reduced LGBTQ+ intergroup anxiety (hypothesis 2); (c) an increased LGBTQ+ intergroup empathy (hypothesis 3). We expected such relationships to be strong enough also after controlling for the quantity of LGBTQ+ contacts and a modern measure of sexual prejudice.

Finally, in Study 3, we wanted to replicate the model and hypotheses of Study 2 by implementing an experimental design in which participants were asked to fulfil an imagined contact task, to imagine a brief encounter with an LGBTQ+ person, either in a neutral way or a positive one.

#### Power and sample sizes

Before starting data collection, to establish an adequate sample size for Study 1, we used an a priori power analysis on the G*Power calculator version 3.1.9.7 (Faul et al., 2009). Results indicated the required sample size to achieve 95% power for detecting a medium effect (*r* = .15), at a significance criterion of *α* = .05, was *N* = 89 for the moderation analysis, including four predictors (i.e., our independent variable, moderating variable, the interaction and the covariate).

The sample size of Studies 2 and 3 were determined through a power analysis designed for a mediation model with a single mediator, performed using an R application (Schoemann et al., 2017). For study 2, we opted for conservative expected effect sizes and number of replications to achieve a robust statistical power (*r* = .15, 1−*β* = .80, replication = 5000, draws = 20,000, Monte Carlo confidence level = 95%). Analysis revealed a minimal sample size of 491 observations for reaching a statistical power of 0.80 (*95% CI* = 0.78, 0.82). The sample size of Study 3 was determined based on the Study 2 results. We opted for conservative expected effect sizes and number of replications to achieve a robust statistical power (.34 < *r <* .56, 1−*β* = .80, replication = 5000, draws = 20,000, Monte Carlo confidence level = 95%). Analysis revealed a minimal sample size of 370 observations for reaching a statistical power of 0.80 (*95% CI* = 0.78, 0.81).

## STUDY 1

Based on the intergroup contact framework, Study 1 was designed to test a moderation model including the quantity of direct contacts with LGBTQ+ people, the quality of such contacts, and their interaction, as predictors of LGBTQ+ conspiracy beliefs. To give more robustness to such results, we investigated such relationships controlling for a modern measure of sexual prejudice (i.e., denial of discrimination), adding this variable as a covariate to the model.

### Method

#### Participants and procedure

Participants were recruited through Prolific, receiving monetary compensation (£7.50/h) with an average completion time of 5–10 min. The online survey aimed to assess some personal characteristics and general political opinions about social and gender issues. Inclusion criteria included (a) heterosexual or predominantly heterosexual sexual orientation; (b) cisgender man or woman gender identity; (b) Italian nationality; (c) at least 18 years old; (d) not failing the attentional check item.

Before proceeding with the questionnaire by Qualtrics, participants read and accepted the informed consent. Subsequently, they responded to socio‐demographic data, and finally, they were presented with the other tools of the questionnaire, including the measures of LGBTQ+ contact, LGBTQ+ conspiracy beliefs, sexual prejudice, and the attentional check item. At the end of the questionnaire, participants were thanked and redirected to the Prolific site for compensation.

Although 261 participants completed the questionnaire, seven participants were removed since they did not identify as cisgender men or cisgender women (i.e., agender, transgender, or other identities), and six participants were removed because they reported a non‐heterosexual sexual orientation. Thus, the final sample included 253 Italian participants (*N*_*Men*_ = 140, 55.3%; *N*_*Women*_ = 113, 44.7%), ranging from 21 to 65 years old (M = 35.04, SD = 10.96). Most of them were atheists (*N* = 165, 65.2%), followed by Catholics (*N* = 76, 30.0%), and by other religions (*N* = 12, 4.8%). There were 108 participants with at most a secondary school diploma (42.7%), whereas those with at least a bachelor's degree or higher were 145 (57.3%). Finally, the sample included students (*N* = 60, 23.7%), workers (*N* = 147, 58.1%), unemployed and retired people (*N* = 34, 13.4%), and respondents who answered ‘other’ (*N* = 12, 4.7%).

##### Measures

The first part of the questionnaire included socio‐demographic questions and political orientation. Please, see Table 1 for descriptives. Participants were invited to report their biological sex (sex assigned at birth), gender identity, age, sexual orientation, nationality, educational level, working status, and political orientation.

**TABLE 1:** Correlations and descriptive (Study 1, *N* = 253).

	Gender	Age	EDU	PO	Contact quantity	Contact quality	Denial	GILC
Gender	1							
Age	.09	1						
EDU	.12	.09	1					
PO	−.07	.16*	.02	1				
Contact quantity	.09	−.03	.10	−.26**	1			
Contact quality	.31**	.08	.04	−.26**	.35**	1		
Denial	−.22**	.08	−.04	.29**	−.20**	−.23**	1	
GILC	−.16*	.11	−.11	.43**	−.22**	−.35**	.48**	1
M	–	35.04	–	3.15	3.21	4.00	2.11	1.68
SD	–	10.96	–	1.28	1.35	0.74	0.88	1.00
*Skewness*	–	0.88	–	0.61	0.10	−0.29	0.80	1.66
*Kurtosis*	–	−0.15	–	−0.04	−1.20	−0.92	0.25	1.97

*Note*: *p < .05; **p < .01; Gender: 1 = Male; 2 = Female.

Abbreviations: Contact quality, Quality of LGBTQ+ contacts with higher scores corresponding to more positive contacts; Contact quantity, Number of LGBTQ+ direct contacts; EDU, Education; GILC, Gender Ideology and LGBTQ+ Lobby Conspiracies Scale; PO, Political orientation: from 1 = extreme left to 7 = extreme right.

Participants could indicate their biological sex by choosing one of three options (i.e., male; female; other), whereas gender identity was asked through eight options (i.e., cisgender man; cisgender woman, transgender FtoM, transgender MtoF, Transgender nonbinary; genderfluid; agender; other). A single item with eight options (Salvati et al., 2019, 2021; Salvati & Chiorri, 2023) was used to investigate participants' sexual orientation (i.e., exclusively heterosexual; predominantly heterosexual; bisexual; predominantly homosexual; exclusively homosexual; pansexual; asexual; other). Furthermore, participants indicated their political orientation through one single item (Salvati et al., 2022) on a 7‐point scale ranging from 1 (*extreme left*) to 7 (*extreme right*).

###### LGBTQ+ contact quantity

The number of participants' direct contacts with LGBTQ+ people was explored through the following single item: ‘How many LGBTQ+ (lesbian, gay, bisexual, trans, queer) people do you?’ Participants could select one of five options: (1 = none; 2 = one or two; 3 = three of four; 4 = five or six; 5 = more than six). The item and the options were very similar to other measures already used in previous studies to investigate intergroup contact quantity (Lolliot et al., 2015; Piumatti & Salvati, 2020; Vezzali et al., 2022; Vezzali & Capozza, 2011; Vezzali & Giovannini, 2012).

###### LGBTQ+ contact quality

The quality of participants' direct contacts with LGBTQ+ people was explored through five bipolar scales, which is a procedure already used in several previous studies (Lolliot et al., 2015; Vezzali et al., 2010, 2022). An introductory item asked: ‘When you have contacts with LGBTQ+ people, how do you perceive these contacts’? Then participants were presented with the five bipolar scales (i.e. hostile/friendly; competitive/cooperative; rude/kind), each one with a 5‐point scale, where one denoted the negative and five the positive pole. The total score was calculated through the mean of the five items so that higher scores corresponded to more positive quality contacts (*α* = .85).

###### LGBTQ+ conspiracy beliefs

Participants responded to the *The Gender Ideology and LGBTQ+ Lobby Conspiracies (GILC) scale* (Salvati, Pellegrini, De Cristofaro, Costacurta, & Giacomantonio, 2024). The tool consists of nine items on a 5‐point Likert scale, ranging from 1 (*totally disagree*) to 5 (*totally agree*). An example item is ‘There are very powerful LGBTQ+ people who manage to influence the decisions of Parliament and the Government, to the detriment of other citizens’. The final GILC score was calculated through the mean of all items (*α* = 0.97).

###### Sexual prejudice

The *denial of discrimination against LGBTQ+ people* (*Denial*; Massey, 2009; Salvati, Pellegrini, De Cristofaro, Costacurta, & Giacomantonio, 2024) is a subtle and modern form of prejudice against LGBTQ+ people that measures people's beliefs that discrimination against LGBTQ+ people is no longer an issue today. It consists of three items on a 5‐point Likert scale, ranging from 1 (*totally disagree*) to 5 (*totally agree*). An example item is ‘Discrimination against LGBTQ+ people is no longer a problem in this country’. The total score was calculated through the mean of the items (*α* = 0.83).

##### Data analyses

Before testing the hypotheses, preliminary correlation analyses were run to test the multicollinearity of all the variables' correlations, which was considered acceptable if correlations were below |.80| (Field, 2009).

Hypotheses were tested through a moderated regression model, including contact quality as the predictor, contact quantity as the moderator, and GILC as the criterion. The model was run with and without denial of discrimination as a covariate. Subsequently, simple slope analyses were conducted to explore the interaction effect further. All the analyses were conducted by SPSS and the vers. 4.0 of its macro PROCESS (Model 1, Hayes, 2017).

### Results/discussion

Preliminary analyses (Table 1) showed that all skewness indices ranged between −0.29 and 1.66, whereas all the kurtosis indexes ranged between −0.92 and 1.97, confirming that all the continuous variables were normally distributed. Correlations showed that multicollinearity was not an issue, since all the values ranged between −0.35 and 0.48.

Correlations gave a first preliminary support to the hypotheses. Indeed, in line with Hypothesis 1, lower contact quantity was associated with higher GILC scores, *r* = −.22, *p* < .01, suggesting that participants who reported fewer LGBTQ+ contacts, the more they were likely to report LGBTQ+ conspiracy beliefs. At the same time, in line with Hypothesis 2, lower contact quality was associated with a higher GILC score, *r* = −.35, *p* < .01, indicating that the fewer participants reported positive contacts with LGBTQ+, the more they were likely to report LGBTQ+ conspiracy beliefs.

Furthermore, the results of the moderated regression model confirmed all three hypotheses (Table 2), explaining a significant proportion of variance, *R* ^2^ = .31, *F*(4, 227) = 25.90, *p* < .001. Before running the moderated regression model, we removed 21 participants who reported having no direct contact with LGBTQ+ people. Thus, such a model was run on 232 participants, rather than the 253 original ones. Corroborating preliminary correlations and confirming Hypothesis 1 and Hypothesis 2, the results showed that lower contact quantity and lower contact quality were related to higher GILC. At the same time, such relations were qualified by the interaction term, which adds a significant proportion of variance, *ΔR* ^2^ = .01, *F*(1, 227) = 4.43, *p* = .036, confirming Hypothesis 3 too.

**TABLE 2:** Moderated multiple regression model for predicting GILC scores, based on Contact quantity and Contact quality (Study 1, *N* = 232).

	*B*	SE	*t*	*p*	*CI*_*95%*_ LLCI	*CI*_*95%*_ ULCI
Constant	4.20	1.00	4.21	<.001	2.235	6.164
Contact quantity	−0.65	0.27	−2.39	.018	−1.193	−0.114
Contact quality	−0.77	0.24	−0.3.29	.001	−1.238	−0.311
Contact quantity × Contact quality	0.14	0.07	2.11	.036	0.009	0.267
Denial	0.43	0.07	6.58	<.001	0.302	0.560

*Note*: 21 participants were removed from the analysis because they reported having no direct contact with LGBTQ+ people.

Abbreviations: Contact quality, Quality of LGBTQ+ contacts with higher scores corresponding to more positive contacts; Contact quantity, Number of LGBTQ+ direct contacts; GILC, Gender Ideology and LGBTQ+ Lobby Conspiracies Scale.

**FIGURE 1:**
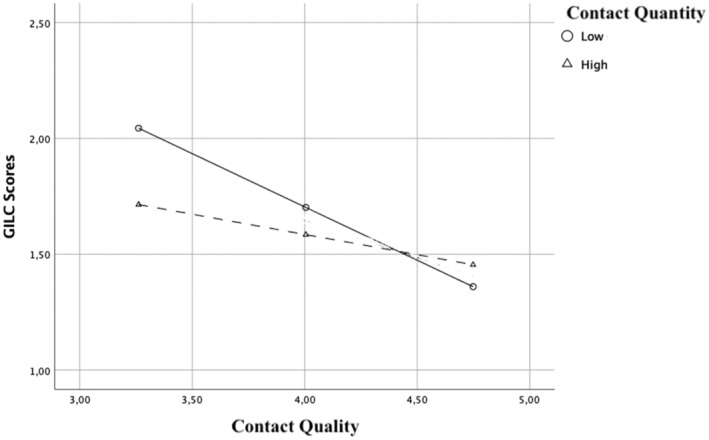
Conditional effect of Contact Quantity on GILC scores, as a function of Contact Quality (Study 1, *N* = 232). Contact Quality, Quality of LGBTQ+ contacts with higher scores corresponding to more positive contacts; Contact Quantity, Number of LGBTQ+ direct contacts; GILC, Gender Ideology and LGBTQ+ Lobby Conspiracies Scale.

Specifically, simple slopes analyses (Figure 1) confirmed that the effect of contact quality on GILC is significant for low levels of contact quantity, *B* = −0.47, SE = 0.11, *t* = −4.26, *p* < .001, *CI*_*95%*_ [−0.684, −0.251], but not for high levels of contact quantity, *B* = −0.14, SE = 0.11, *t* = −1.25, *p* = .214, *CI*_*95%*_ [−0.363, −0.082]. Such results suggest that when people have a lot of contacts with LGBTQ+ people, the quality of such contacts might be less influential in predicting LGBTQ+ conspiracy beliefs, probably because a high number of LGBTQ+ contacts is more likely to be associated with better quality too. Instead, when people report few contacts with LGBTQ+ people, the quality of such contacts might have a greater impact on LGBTQ+ conspiracy beliefs, indicating that people with lower‐quality contact with LGBTQ+ people reported higher LGBTQ+ conspiracy beliefs, compared with people with better contact quality with LGBTQ+ people.

All the results were significant, controlling for a modern measure of sexual prejudice (Denial) too, which was added to the model as a covariate. This gave stronger robustness to our results, suggesting that the quantity and the quality of direct contacts with LGBTQ+ people can provide a specific contribution to explaining the differences in people's adherence to LGBTQ+ conspiracy beliefs, independent of their level of sexual prejudice, which previous literature has already found to be related to LGBTQ+ conspiracy beliefs (Salvati, Pellegrini, De Cristofaro, Costacurta, & Giacomantonio, 2024).

Study 1 pointed out that both quantity and quality of contact were directly associated with lower levels of LGBTQ+ conspiracy beliefs. However, results suggested that intergroup contact was particularly beneficial for participants with fewer but more positive contacts, showing that they also reported lower LGBTQ+ conspiracy beliefs.

## STUDY 2

Moving on from the results of Study 1, a further step was taken to explore the potential processes that might contribute to explaining the relationship between the higher quality of LGBTQ+ contacts and the lower adherence to LGBTQ+ conspiracy beliefs. Based on the theoretical framework of contact theory (Allport, 1954) and its robust empirical evidence (Kanamori et al., 2022; Paolini et al., 2004; Vezzali & Giovannini, 2012; Vezzali, Lolliot et al., 2023; Vezzali, Trifiletti, et al., 2023; Zhou et al., 2019), we selected the three main mediators explored in previous literature on the relationship between intergroup contact and prejudice, namely, knowledge about the outgroup, intergroup anxiety, and intergroup empathy (Pettigrew & Tropp, 2006, 2008).

### Method

#### Participants and procedure

Like Study 1, participants were recruited through Prolific and received monetary compensation (£6.00/h). The procedure was the same as in Study 1 as well as the inclusion criteria, except for the nationality. Indeed, considering the low frequency of Italians on Prolific, we preferred to recruit participants from the UK who represent the majority of Prolific users.

Although 554 participants completed the questionnaire, eight participants were removed since they did not identify as cisgender men or cisgender women, and 16 participants were removed because they reported a non‐heterosexual sexual orientation. Thus, the final sample included 512 participants (*N*_*Men*_ = 253, 49.4%; *N*_*Women*_ = 259, 50.6%), ranging from 18 to 77 years old (M = 47.27, SD = 14.05). Most of them were atheists (*N* = 309, 60.4%), followed by Protestants/Anglicans (*N* = 95, 18.6%), Catholics (*N* = 66, 12.9%), and other religions (*N* = 42, 8.2%). There were 228 participants with at most a secondary school diploma (44.5%), whereas those with at least a bachelor's degree or higher were 284 (55.5%). Finally, the sample was made up of students (*N* = 22, 4.3%), workers (*N* = 345, 67.4%), unemployed and retired people (*N* = 119, 23.2%), and respondents who answered ‘other’ (*N* = 26, 5.1%).

##### Measures

All the socio‐demographic questions were the same as used in Study 1, as well as the measures of LGBTQ+ Contact Quantity and Quality, the Gender ideology and LGBTQ+ Lobby conspiracies (GILC) scale (Salvati, Pellegrini, De Cristofaro, Costacurta, & Giacomantonio, 2024; *α* = .96), and the denial of discrimination of LGBTQ+ people (Massey, 2009; Salvati, Pellegrini, De Cristofaro, Costacurta, & Giacomantonio, 2024; *α* = .83).

###### LGBTQ+ group knowledge

Knowledge about the LGBTQ+ group was measured through six items formulated for the current research project and inspired by several previous works (i.e., Clark & Kosciw, 2022). Items asked participants to rate on a 5‐point Likert scale from 1 = *not at all*, to 5 = *completely*, how much they believe they knew about several issues and characteristics about LGBTQ+ people and community. The items were introduced by the sentence ‘How much do you think you know…’, and then the items were presented (‘…the difference between “sexual orientation” and “gender identity”’; ‘…the meaning of the acronym LGBTQ+’; ‘…the stereotypes relating to LGBTQ+ people’; ‘…the history of the LGBTQ+ movement and community’; ‘…what are the LGBTQ+ national and international associations’; ‘…scientific data on LGBTQ+ people (e.g. well‐being and development of children raised by same‐gender couples)’). The average score of all the items was calculated as total score, so that higher scores in this scale corresponded to higher LGBTQ+ group knowledge (*α* = .80).

###### LGBTQ+ intergroup anxiety

To measure participants' LGBTQ+ group anxiety, the procedure by Stephan and Stephan (1985) was adapted for the current research. Firstly, participants were asked to imagine a social interaction with LGBTQ+ people. Specifically, they read the following text: ‘Now, we ask you to imagine yourself in the following scene: You are in a social situation such as a dinner or a work project with a group of people. At a certain moment, you start talking about sexual orientation. All the other participants express a different sexual orientation from yours. Thus, if you were the only heterosexual member in the group and you were interacting with LGBTQ+ people, how would you feel compared to occasions when you are interacting with heterosexual people group?’ At this point, participants were invited to rate six affective states on a 5‐point Likert scale from 1 = *not at all*, to 5 = *extremely*. The emotions included happy, awkward, self‐conscious, confident, relaxed, and defensive (Lolliot et al., 2015; Paolini et al., 2004). The total score was calculated by averaging the six scores, after reversing three items (i.e. happy, confident, and relaxed), so that higher scores corresponded to higher LGBTQ+ intergroup anxiety (*α* = .87).

###### LGBTQ+ intergroup empathy

This measure was investigated by asking participants to think about LGBTQ+ people, not one in particular, but their social group more generally. Thus, using a 5‐point Likert scale from 1 = not at all, to 5 = extremely, they were invited to respond to four items introduced by the following: ‘When you think about LGBTQ+ people, to what extent…’. The items were ‘Do you feel in tune with them?’; ‘Do you feel you share their emotions?’; ‘Do you understand their feelings?’; ‘Do you share their joys and sorrows?’ (Capozza et al., 2013). The final score corresponded to the average score of the four items, so that higher scores corresponded to higher LGBTQ+ intergroup empathy (*α* = .91).

#### Data analyses

As for Study 1, preliminary correlation analyses were run. Subsequently, we tested our hypotheses through a mediational model, including contact quality as predictor, GILC as criterion, and LGBTQ+ Group Knowledge, LGBTQ+ Intergroup Anxiety, and LGBTQ+ Intergroup Empathy as mediators. The model was run both including and not including denial of discrimination and contact quantity as covariates. All the analyses were conducted by SPSS and the vers. 4.0 of its macro PROCESS (Model 4, Hayes, 2017).

### Results/discussion

Preliminary analyses (Table 3) showed that multicollinearity was not an issue, since all the values ranged between −0.56 and 0.56. Correlations corroborated the results of study 1, showing that lower contact quality was associated with higher GILC score, *r* = −.41, *p* < .01, indicating that the participants who reported fewer positive contacts with LGBTQ+, the more they were likely to report LGBTQ+ conspiracy beliefs. At the same time, correlations provided initial support for our hypotheses, because higher levels of contact quality were associated with higher levels of LGBTQ+ group knowledge, *r* = .18, *p* < .01, to lower levels of LGBTQ+ intergroup anxiety, *r* = −.56, *p* < .01, and to higher levels of LGBTQ+ intergroup empathy, *r* = .56, *p* < .01. Higher levels in GILC were associated with higher levels of intergroup anxiety, *r* = .34, *p* < .01, and with lower levels of LGBTQ+ intergroup empathy, *r* = −.42, *p* < .01. However, the correlations showed that GILC and LGBTQ+ Knowledge were not associated, *r* = −.07, *p* > .05.

**TABLE 3:** Correlations and descriptive (Study 2, *N* = 512).

	Gender	Age	EDU	PO	Contact quantity	Contact quality	Knowledge	Anxiety	Empathy	Denial	GILC
Gender	1										
Age	.02	1									
EDU	.06	−.11*	1								
PO	−.11*	.17**	−.14**	1							
Contact quantity	.10*	−.11*	.22**	−.28**	1						
Contact quality	.23**	.01	.11*	−.17**	.23**	1					
Knowledge	.04	−.22**	.13**	−.19**	.28**	.18**	1				
Anxiety	−.12**	−.03	−.03	.20**	−.29**	−.56**	−.23**	1			
Empathy	.25**	−.05	.07	−.32**	.34**	.56**	.32**	−.56**	1		
Denial	−.12**	.12**	−.12**	.36**	−.18**	−.19**	−.09	.20**	−.21**	1	
GILC	−.14**	.09*	−.17**	.42**	−.29**	−.41**	−.07	.34**	−.42**	.42**	1
M	–	47.24	–	3.54	3.15	4.12	2.98	2.53	3.06	2.63	2.28
SD	–	14.04	–	1.32	1.33	0.83	0.68	0.59	0.95	0.95	1.12
*Skewness*	–	−0.17	–	0.24	0.13	−0.69	−0.05	0.46	−0.49	0.37	0.55
*Kurtosis*	–	−0.92	–	−0.59	−1.20	−0.16	0.20	0.11	−0.16	−0.30	−0.76

*Note*: *p < .05; **p < .01; Gender: 1 = Male; 2 = Female.

Abbreviations: Contact quality, Higher scores correspond to more positive LGBTQ+ contacts; Contact quantity, Number of LGBTQ+ direct contacts; EDU, Education; GILC, Gender Ideology and LGBTQ+ Lobby Conspiracies Scale; PO, Political orientation: from 1 = extreme left to 7 = extreme right.

**FIGURE 2:**
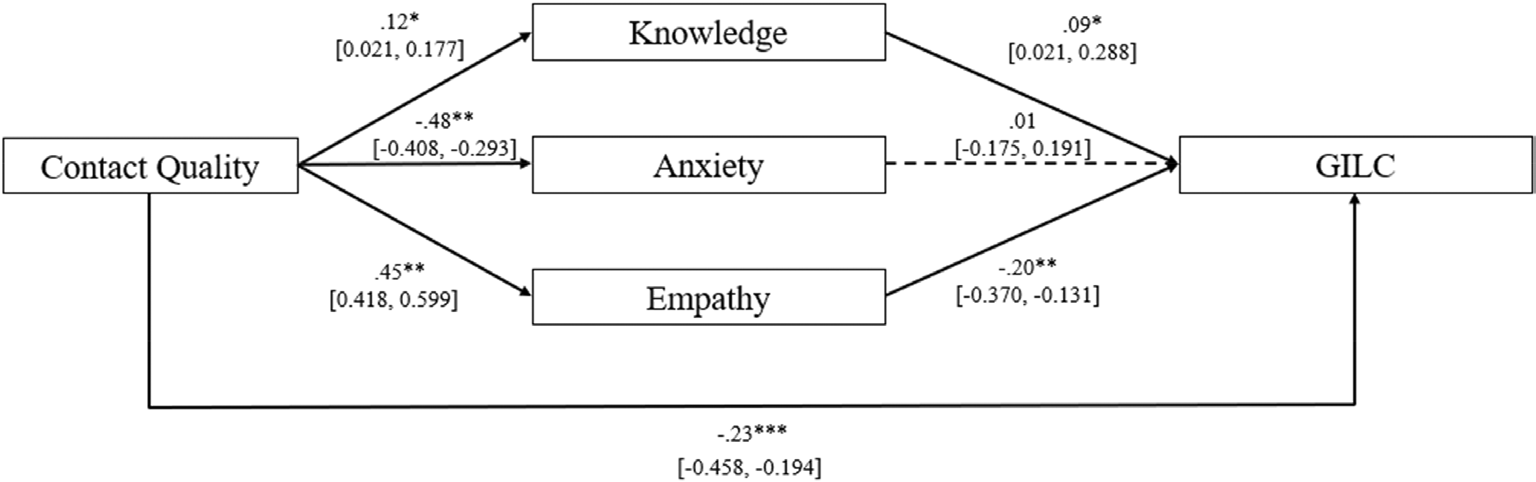
Mediational model (Study 2: *N* = 463). *p < .05; **p < .01; Dotted lines indicate non‐significant relationships; Standardized *β* are shown. Contact Quality, Higher scores correspond to more positive LGBTQ+ contacts; GILC, Gender Ideology and LGBTQ+ Lobby Conspiracies Scale.

Furthermore, the results of the mediational model partially confirmed our hypotheses (Table 4; Figure 2), explaining a significant proportion of variance in GILC, *R* ^2^ = .29, *F*(3, 459) = 62.90. *p* < .001. Before running the mediational model, we removed 49 participants who reported having no direct contact with LGBTQ+ people. Thus, such a model was run on 463 participants, rather than the 512 original ones. The negative association between contact quality and GILC remained significant after adding the three mediators to the model, suggesting a partial mediational role of them. Specifically, the results showed that higher levels of contact quality were associated with lower levels of GILC, through high levels of LGBTQ+ group knowledge (hypothesis 1), through high levels of LGBTQ+ intergroup empathy (hypothesis 3), but not through low levels of LGBTQ+ intergroup anxiety (hypothesis 2). Indeed, on the one hand, higher levels of contact quality were related to higher levels of LGBTQ+ group knowledge, lower levels of LGBTQ+ intergroup anxiety, and higher levels of LGBTQ+ intergroup empathy. On the other hand, only higher levels of LGBTQ+ intergroup empathy were associated with lower levels of GILC, whereas LGBTQ+ intergroup anxiety was not associated with GILC, and contrary to the hypotheses and what emerged from the correlations, LGBTQ+ group knowledge was marginally, but positively, associated with GILC. However, the indirect association between contact quality and GILC via LGBTQ+ intergroup empathy was stronger than the one via LGBTQ+ group knowledge, *ΔB* = 0.12, SE = 0.03, *CI*_*95%*_ [−0.063, −0.185], suggesting that among the three potential mediators, the LGBTQ+ intergroup empathy plays the primary role in explaining the relationship between contact quality and GILC.

**TABLE 4:** Associations of the mediational model (Study 2: *N* = 463).

DV	IV	*β*	SE	*t*	*p*	95% CI lower	95% CI upper
Knowledge	Contact quality	.12	0.04	2.51	.013	0.021	0.177
Anxiety	Contact quality	−.48	0.03	−11.94	<.001	−0.408	−0.293
Empathy	Contact quality	.45	0.05	11.07	<.001	0.418	0.599
GILC	Contact quality	−.23	0.07	−4.84	<.001	−0.458	−0.194
	Knowledge	.09	0.07	2.27	.024	0.021	0.288
	Anxiety	.01	0.09	0.08	.933	−0.175	0.191
	Empathy	−.20	0.06	−4.11	<.001	−0.370	−0.131
**Indirect associations**							
		***β***	**BootSE**			**BootLLCI**	**BootULCI**
Contact Quality ➔ Knowledge ➔ GILC		.01	0.01			0.001	0.026
Contact Quality ➔ Anxiety ➔ GILC		−.01	0.02			−0.049	0.044
Contact Quality ➔ Empathy ➔ GILC		−.09	0.03			−0.146	−0.040
Total		−.08	0.03			−0.141	−0.026

*Note*: 49 participants were removed from the analysis because they reported having no direct contact with LGBTQ+ people; Standardized *β* are showed; Number of LGBTQ+ contacts (Contact Quantity) and Denial of discrimination (Denial) were added to the model as covariates. *R* ^2^ = .29. *F*(3, 459) = 62.90, *p* < .001. The total effect of Contact Quality on GILC (before the mediators were added in the model): *β* = −.31, SE = .06, *t* = −7.73, *p* < .001, *CI*_*95%*_ [−0.552; −0.329].

Abbreviations: Contact quality, Quality of LGBTQ+ contacts with higher scores corresponding to more positive contacts; GILC, Gender Ideology and LGBTQ+ Lobby Conspiracies Scale.

Thus, the results of Study 2 partially confirmed our expectations: while empathy was positively associated with contact quality and negatively associated with LGBTQ+ conspiracy beliefs, intergroup knowledge showed a positive association with LGBTQ+ conspiracy beliefs, whereas anxiety did not show a significant relationship with LGBTQ+ conspiracy beliefs. Regarding the last unexpected result, one can speculate that unlike prejudice and intergroup relations, which are the main variables on which contact theory has focused on as criterion variables (Pettigrew & Tropp, 2006), conspiracy beliefs might be elicited by other intergroup emotions such as anger, which could be a better mediator rather than anxiety of the relationship between contact quality and GILC, but further studies might verify this speculation.

## STUDY 3

In Study 3, we wanted to experimentally replicate the model and test the hypotheses of Study 2 by implementing an imaginative task consisting of a hypothetical (positive vs. neutral) interaction with an LGBTQ+ person. Thus, we investigated whether the (positive) quality of intergroup contact indirectly affects LGBTQ+ conspiracy beliefs via the mediation of increased knowledge about the outgroup; the reduced intergroup anxiety, and the increased intergroup empathy (Pettigrew & Tropp, 2006, 2008).

### Method

#### Participants and procedure

We ran a third online experiment via Prolific Academic. Like Study 1 and Study 2, participants were recruited through Prolific and received monetary compensation (£6.00/h). The procedure was the same as in Study 2 as well as the inclusion criteria, except for the nationality. In this study, we included both UK and US participants to broaden the pool of potential native English‐speaking respondents on Prolific.

Although there were 547 participants who completed the questionnaire, six participants were removed because they were not cisgender men or cisgender women, and 12 participants were removed because they reported a non‐heterosexual sexual orientation. Thus, the final sample included 529 participants (*N*_*Men*_ = 233, 44.0%; *N*_*Women*_ = 296, 56.0%), ranging from 18 to 87 years old (M = 47.83, SD = 14.82). Most of them were atheists (*N* = 275, 52.0%), followed by Protestants/Anglicans (*N* = 94, 17.8%), Catholics (*N* = 89, 16.8%), and by other religions (*N* = 72, 13.4%). There were 205 participants with at most a secondary school diploma (38.8%), whereas those with at least a bachelor's degree or higher were 324 (61.2%). Finally, the sample was made up of students (*N* = 45, 8.5%), workers (*N* = 354, 66.9%), unemployed and retired people (*N* = 100, 18.9%), and respondents who answered ‘other’ (*N* = 30, 5.7%).

##### Measures

All the socio‐demographic questions were the same as those used in Study 1 and Study 2, as well as the measure of LGBTQ+ Contact Quantity. Subsequently, participants were randomly assigned either to the experimental group (*N* = 256) or to the control group (*N* = 273), following the same procedure by Stathi and Crisp (2008). Particularly, we compared two conditions, namely, an imagined neutral contact and a positive one. Following the rationale of Stathi and Crisp (2008), our aim was to investigate whether lower levels of LGBTQ+ conspiracy beliefs were specifically related to positive imagined contact. Furthermore, despite several works investigating imaged intergroup contact including a control condition (e.g. no contact with a member of the outgroup), this comparison did not inform us whether the valence of the contact itself could be more beneficial and overall, the control condition with which imagined contact was compared did not significantly influence effect sizes of the results (see Miles & Crisp, 2014 for a review).

In both conditions, participants were involved in an imaginative task, inviting them to interact with an LGBTQ+ person. The first part of the instruction was the same for both groups: ‘Please, spend 5 min imagining that you speak to an LGBTQ+ person who has sat next to you on the train. You spend about 30 min chatting about several things, until you reach your stop and depart the train’. At this point, the instructions for the control group ended, while those for the experimental group continued as follows: ‘During the conversation, you find out some interesting and positive things about them’. In the end, both groups were invited to respond to one open question asking to list the things they found out about that person.

As a manipulation check, we used a single item structured as a slider that the participant could drag on a percentage scale from 0 to 100, asking the following: ‘Of all the things that came to mizsand when thinking about this person, what percentage were positive?’, M = 85.3, SD = 18.6.

After the manipulation check, participants responded to the same measure of LGBTQ+ Group Knowledge (*α* = .81) and *LGBTQ+ Intergroup Empathy* (Capozza et al., 2013; *α* = .91) used in Study 2. At the same time, the measure of the *LGBTQ+ Intergroup Anxiety* was simplified compared with one used in Study 2, because the imaginative social dinner task was removed. Thus, participants were asked to respond to four items (i.e. ‘Worried’; ‘Nervous’; ‘Uncomfortable’; ‘Anxious’), on a 5‐point Likert scale from 1 = *not at all*, to 5 = *extremely*, after presenting the following question ‘How do you feel when you think about interacting with LGBTQ+ people?’ (Lolliot et al., 2015; Paolini et al., 2004; Stephan & Stephan, 1985; *α* = .91).

The *Gender ideology and LGBTQ+ Lobby conspiracies (GILC) scale* (Salvati, Pellegrini, De Cristofaro, Costacurta, & Giacomantonio, 2024; *α* = .96), and the *denial of discrimination against LGBTQ+ people* (Massey, 2009; Salvati, Pellegrini, De Cristofaro, & Giacomantonio, 2024; *α* = .83) were the same as those used in Study 2.

###### Data analyses

As for Studies 1 and 2, we tested normality and multicollinearity through kurtosis, skewness and correlation analyses. Subsequently, we verified the effectiveness of our manipulation testing the differences between the experimental and control groups through an ANOVA, with the manipulation check item as the dependent variable. Afterwards, we tested the effect of our manipulation on the three potential mediators of the hypothesized model to determine which mediators to include in the model to test our hypotheses. The hypothesized model would have the experimental condition of positive (vs. neutral) contact with a LGBTQ+ person as the predictor, the knowledge, the anxiety and empathy about LGBTQ+ group as parallel mediators, and GILC as the criterion (Model 4, Hayes, 2017). The model was run both including and not including the number of LGBTQ+ contacts, the denial of discrimination and variables for which the manipulation was not effective, as covariates.

### Results/discussion

Preliminary analyses (Table 5) showed that multicollinearity was not an issue, since all the values ranged between −0.49 and 0.45. Higher levels in GILC were associated with higher levels of intergroup anxiety, *r* = .45, *p* < .01, with lower levels of LGBTQ+ intergroup empathy, *r* = −.36, *p* < .01, and with lower levels of LGBTQ+ Knowledge, *r* = −.16, *p* < .01.

**TABLE 5:** Correlations and descriptive (Study 3, *N* = 529).

	Gender	Age	EDU	PO	Contact quantity	GROUPS	Knowledge	Anxiety	Empathy	Denial	GILC
Gender	1										
Age	.02	1									
EDU	.07	−.08	1								
PO	−.13**	.13**	−.15**	1							
Contact quantity	.18**	−.12**	.20**	−.25**	1						
GROUPS	.07	.01	.01	−.04	−.02	1					
Knowledge	.07	−.16**	.14**	−.21**	.21**	.01	1				
Anxiety	−.21**	−.08	−.14**	.30**	−.29**	−.01	−.20**	1			
Empathy	.19**	.02	.08	−.36**	.30**	.10*	.33**	−.49**	1		
Denial	−.13**	.14**	−.04	.37**	−.23**	.03	−.08	.18**	−.18**	1	
GILC	−.05	.05	−.06	.40**	−.26**	.04	−.16**	.45**	−.37**	.44**	1
M	–	42.83	–	3.55	3.23	–	3.03	1.49	3.29	2.70	2.26
SD	–	14.82	–	1.28	1.35	–	0.74	0.81	0.98	0.99	1.10
*Skewness*	–	0.33	–	0.35	0.09	–	−0.19	1.89	−0.50	0.33	0.58
*Kurtosis*	–	−0.81	–	−0.56	−1.34	–	0.12	3.30	−0.16	−0.50	−0.59

*Note*: *p < .05; **p < .01; Gender: 1 = Male; 2 = Female.

Abbreviations: Contact quantity, Number of LGBTQ+ direct contacts; EDU, Education; GILC, Gender Ideology and LGBTQ+ Lobby Conspiracies Scale; GROUPS, Experimental Group = 1; Control Group = 0; PO, Political orientation: from 1 = extreme left to 7 = extreme right.

The ANOVA conducted on the manipulation check item confirmed that our manipulation was effective. Indeed, the experimental group reported a higher percentage of positive thoughts that came to mind during the imaginative interaction with a LGBTQ+ people, M = 87.99; SD = 15.83, compared with the control group, M = 82.81; SD = 20.66, *F*(1, 527) = 10.38, *p* = .001.

The three ANOVAs conducted on the potential mediators of the model showed a significant difference between the experimental and control group on LGBTQ+ Intergroup Empathy only, *F*(1, 527) = 5.74, *p* = .017, confirming that the experimental group reported higher LGBTQ+ Intergroup Empathy, M = 3.39; SD = 0.96, compared with the control group, M = 3.19; SD = 0.99. Contrary to our expectations, the analyses did not find significant differences in LGBTQ+ Group Knowledge, *F*(1, 527) = 0.01, *p* = .995, M_tot_ = 3.03, SD_tot_ = 0.74, nor on LGBTQ+ intergroup anxiety, *F*(1,527) = 0.10, *p* = .748, M_tot_ = 1.49, SD_tot_ = 0.81.

Considering the previous results, we decided to test a mediational model where the experimental condition of positive (vs. neutral) contact with a LGBTQ+ person was the predictor, the LGBTQ+ intergroup empathy was the mediator, and the GILC was the criterion (Model 4, Hayes, 2017), whereas the number of LGBTQ+ contacts, the denial of discrimination, the LGBTQ+ group knowledge, and the LGBTQ+ intergroup anxiety were entered as covariates.

**FIGURE 3:**
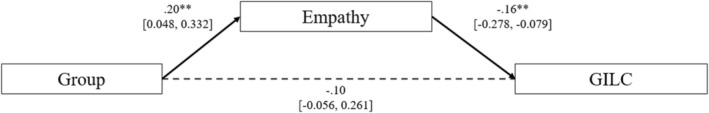
Mediational model (Study 3: *N* = 486). **p < .01; Dotted lines indicate non‐significant relationships; Standardized *β* are shown. GILC, Gender Ideology and LGBTQ+ Lobby Conspiracies Scale; GROUPS, Experimental Group = 1; Control Group = 0.

The results of the mediational model partially confirmed the expected predictions (Table 6; Figure 3), explaining a significant proportion of variance in GILC, *R* ^2^ = .33, *F*(6, 479) = 39.05, *p* < .001. Before running the mediational model, we removed 43 participants who reported having no direct contact with LGBTQ+ people. Thus, such a model was run on 486 participants, rather than the 529 original ones. Although the direct effect of our manipulation on GILC, before and after the mediator was added to the model, was not significant, the results showed that participants in the experimental group, rather than the control group, reported higher levels of LGBTQ+ intergroup empathy, which in turn were associated with lower GILC scores. Furthermore, the indirect effect of experimental conditions on GILC via LGBTQ+ empathy was significant. The results were confirmed, both including and not including the covariates.

**TABLE 6:** Associations of the Mediational Model (Study 3: *N* = 486).

DV	IV and covariates	*β*	SE	*t*	*p*	95% CI lower	95% CI upper
Empathy	GROUPS	.20	0.07	2.63	.009	0.048	0.332
	Contact Quantity	.09	0.03	2.19	.029	0.007	0.129
	Denial	−.09	0.04	−2.12	.034	−0.153	−0.006
	Knowledge	.22	0.05	5.58	<.001	0.192	0.400
	Anxiety	−.36	0.05	−8.92	<.001	−0.556	−0.355
GILC	GROUPS	.10	0.08	1.27	.205	−0.056	0.261
	Empathy	−.16	0.05	−3.53	<.001	−0.278	−0.079
	Contact Quantity	−.07	0.03	−1.80	.073	−0.130	−0.006
	Denial	.36	0.04	9.21	<.001	0.302	0.466
	Knowledge	−.02	0.06	−0.48	.630	−0.149	0.090
	Anxiety	.24	0.06	5.61	<.001	0.223	0.463
**Indirect effect**		***β***	**BootSE**			**BootLLCI**	**BootULCI**
GROUPS ➔ Empathy ➔ GILC		−.03	0.01			−0.069	−0.005

*Note*: 43 participants were removed from the analysis because they reported having no direct contact with LGBTQ+ people; Standardized *β* are showed; *R* ^2^ = .33. *F*(6, 479) = 39.05, *p* < .001. The total effect of GROUPS on GILC (before the mediator was added in the model): *β* = .06, SE = .08, *t* = 0.84, *p* = .399, *CI*_*95%*_ [−0.091; 0.228].

Abbreviations: Contact quantity, Number of LGBTQ+ contacts; GILC, Gender Ideology and LGBTQ+ Lobby Conspiracies Scale; GROUPS, Experimental Group = 1; Control Group = 0.

To summarize, Study 3 experimentally tested whether positive intergroup contact and LGBTQ+ conspiracy beliefs could be indirectly associated via increased LGBTQ+ knowledge, reduced intergroup anxiety and higher intergroup empathy. Results supported the indirect role of empathy in the relationship between LGBTQ+ conspiracy beliefs and intergroup contact, however, no other significant indirect effects were found.

## FINAL DISCUSSION

The present research stemmed from previous findings on intergroup contact (Allport, 1954; Pettigrew & Tropp, 2008) by investigating whether the quality and quantity of contact are related to the endorsement of conspiracy beliefs against LGBTQ+. Since conspiracy beliefs might be extremely difficult to change directly (Jolley & Douglas, 2014, 2017; Sunstein & Vermeule, 2009), finding strategies to lessen (LGBTQ+) conspiracy beliefs is crucial (Brown, 2005): intergroup contact may represent one of these strategies (Jolley et al., 2023).

Study 1 showed that both quantity and quality of contact were directly associated with lower levels of LGBTQ+ conspiracy beliefs. However, results seemed to hint that intergroup contact was more beneficial for participants reporting fewer but more positive contact since they also showed lower LGBTQ+ conspiracy beliefs. One can speculate that despite our society changing and becoming more complex, people do not completely take advantage of the full (prejudice‐reducing) benefits of intergroup contact since opportunities for contact are often not taken up, and segregation persists (Paolini et al., 2018). Hence, people who hold higher conspiracy beliefs could be more likely to avoid interactions with members of the outgroup in their everyday lives and thus the quality of such contacts might be more beneficial in producing positive outcomes.

Taking a step forward, Studies 2 and 3 investigated the main factors usually associated with the reduction of intergroup prejudice via intergroup contact (Pettigrew & Tropp, 2008) on LGBTQ+ conspiracy beliefs by implementing respectively cross‐sectional and experimental designs. Specifically, we investigated whether LGBTQ+ knowledge, intergroup anxiety and intergroup empathy could be indirectly associated with positive intergroup contact and LGBTQ+ conspiracy beliefs. Results of Study 2 partially confirmed our expectations: while empathy was positively associated with contact quality and negatively associated with LGBTQ+ conspiracy beliefs, intergroup knowledge showed a positive association with LGBTQ+ conspiracy beliefs, whereas anxiety did not show a significant relationship with LGBTQ+ conspiracy beliefs. Since Study 2 pointed out the key role of intergroup empathy, Study 3 focus revolved around the relationship between positive intergroup contact and conspiracy beliefs via intergroup empathy. Findings pointed out a full mediation with positive contact associated with lower conspiracy beliefs against LGBTQ+ people, through intergroup empathy.

Findings suggested that intergroup empathy might play a fundamental role in the endorsement of LGBTQ+ conspiracy beliefs. In line with previous work (e.g. Kanamori et al., 2022) they pointed out the importance of emotional engagement in reducing negative beliefs against disadvantaged groups. Indeed, empathy has been shown to facilitate emotional understanding of outgroup members and reduce prejudice and stereotypes by also promoting outgroup humanization (Capozza et al., 2013). Contrary to our hypotheses, higher LGBTQ+ knowledge did not play a substantial role concerning LGBTQ+ conspiracy beliefs, displaying only a marginal association in Study 2 and no association in Study 3. These unexpected findings seem to suggest that the mere acquisition of knowledge concerning LGBTQ+ people may not suffice to combat conspiracy beliefs against this community. This pattern may be related to the fact that conspiracy beliefs are difficult to change directly, therefore participants who hold conspiratorial views may interpret new information about LGBTQ+ in a biased way coherently with their conspiratory narrative. This is in line with research concerning motivated reasoning which states that individuals tend to process information in a way that usually confirms their existing worldview (Kunda, 1990). Furthermore, these results could be related to the measurement of intergroup knowledge we adopted. Indeed, our measure referred to the mere ‘perception’ of knowing the outgroup which per sè does not translate into a better (i.e., more correct) knowledge of LGBTQ+ people.

Finally, while LGBTQ+ intergroup anxiety was significantly associated with positive intergroup contact, it was not once the indirect associations with LGBTQ+ conspiracy beliefs were considered. These results are in line with Fox and Williams (2023) who did not find a significant estimated effect of anxiety on conspiracy beliefs. One of the possible explanations is that intergroup anxiety might be a more marginal factor explaining the endorsement of conspiracy beliefs. For instance, Szymanski et al. (2024) suggested that other emotions, such as anger, may play a more central role in tackling conspiracy beliefs. This is also consistent with research demonstrating a positive association between externalizing behaviours (e.g., aggression and propensity for violence) and conspiracy beliefs (Jolley et al., 2023; Marchlewska et al., 2019).

### Practical impact

Overall, the current study has important practical implications. First of all, it highlighted that (positive) intergroup contact could represent a promising and transversal strategy that could be used to tackle conspiracy beliefs against LGBTQ+ people. Furthermore, our findings seem to hint at a prevalence of affective (i.e. empathy and anxiety) over cognitive (i.e. knowledge) processes, by also underlying the crucial role played by empathic concern in reducing conspiracy beliefs against LGBTQ+ people. Hence, training people in socio‐emotional skills may be a key factor in supporting positive intergroup relations and could also constitute a protective element towards conspiracy beliefs, especially for those with less positive contact experiences (Reimer et al., 2021).

Furthermore, researchers have stressed the importance of practical and policy‐relevant work on intergroup contact moving beyond theoretical and laboratory‐based studies to naturalistic settings (Paluck et al., 2019; White et al., 2015). Along this line, some effective attempts have been made during the last decades by implementing intergroup contact programmes in ecological settings. For instance, positive intergroup contact was effective in enhancing trusting of the outgroup and more willing to cooperate with them via listening to a radio soap opera (Paluck, 2009), via workshop (Malhotra & Liyanage, 2005), but also via online direct collaborative programmes (Schumann & Moore, 2022) and indirect online contact (Amzalag & Shapira, 2021). Hence, since conspiracy beliefs and prejudice seem to tap into similar processes, positive intergroup contact may represent a promising strategy to implement effective programmes in reducing conspiracy beliefs too.

## LIMITATIONS AND FUTURE DIRECTIONS

The present research should be considered in light of its strengths and shortcomings, which suggest directions for future research. First, the cross‐sectional design of Studies 1 and 2 limits our ability to draw causal inferences from the data, while the experimental design of Study 3 provided some preliminary evidence concerning the role of intergroup contact in indirectly reducing LGBTQ+ conspiracy beliefs, longitudinal studies or experimental designs would be fundamental to investigate causal effects and their patterns over time. Furthermore, Study 2's results regarding intergroup knowledge raise concerns about whether knowledge is interpreted and used in intergroup contexts about LGBTQ+ conspiracy beliefs. The unexpected results related to intergroup knowledge (i.e. the higher knowledge the higher the conspiracy beliefs) can be related to the self‐report measurement of intergroup knowledge we adopted. Indeed, our measure referred to the mere individual ‘perception’ of knowing the outgroup which per sè does not translate into a better (i.e. more correct) knowledge of LGBTQ+ people. People with less knowledge about a phenomenon tend to overestimate it and feel overtly optimistic about their lack of insight into their own errors (Ehrlinger et al., 2008). Hence, future study could address this issue by adopting a more objective measurement concerning (the nature and framing of) knowledge about the outgroup, as well as individual variations in cognitive processing. Another aspect deserving attention is related to the fact that intergroup contact refers to a broad concept. Literature showed that indirect intergroup contact has the potential to improve intergroup relations, such as extended, vicarious and online ones (Imperato et al., 2021; White et al., 2021). Future research could address the LGBTQ+ conspiracy beliefs endorsement by extending the present investigation and exploring other forms of contact.

Finally, despite our interest revolved around intergroup empathy, knowledge and anxiety, other affective and cognitive factors might play a role in shaping people's conspiracy theories endorsements. Future research could expand the range of mediators to gain a more comprehensive understanding of the phenomenon, for instance, Szymanski et al. (2024) pointed out the interconnected relationship between emotions and beliefs and pointed out the positive associations between anger and the endorsement of general and specific (e.g. COVID‐19) conspiracy beliefs.

## CONCLUSION

In conclusion, the present studies contribute to advancing our understanding of processes related to LGBTQ+ conspiracy beliefs endorsement from an intergroup contact perspective. Findings suggest that while LGBTQ+ conspiracy beliefs present some overlap with factors involved in decreasing prejudice, the pathway to reducing conspiracy beliefs might be slightly different. Our research might offer useful insights which can guide social policies and interventions. Identity the factors that contribute to the endorsement of LGBTQ+ conspiracy beliefs, as well as their consequences on people and communities, may help tailor initiatives to advance social cohesion, tackle prejudice, and support LGBTQ+ people.

## AUTHOR CONTRIBUTIONS

**Sara Panerati:** Formal analysis; writing – original draft; methodology; writing – review and editing. **Marco Salvati:** Conceptualization; data curation; formal analysis; writing – original draft; methodology; supervision; writing – review and editing; funding acquisition.

## CONFLICT OF INTEREST STATEMENT

The authors declare that they have no conflict of interest.

## ETHICS STATEMENT

All the studies of the current research complied with the WMA Declaration of Helsinki (1964/2013), and they were approved by the Ethics Review Board for Research in Psychology at the University of Verona (prot. n. 283385 of 08/07/2024).

## Data Availability

The data that support the findings of this study are available from the corresponding author upon reasonable request.
